# No, either or both parents with metabolic syndrome: comparative study of its impact on sons and daughters

**DOI:** 10.3389/fendo.2025.1518212

**Published:** 2025-04-17

**Authors:** Jun-Hong Park, Min Hyung Cho, Hae Sang Lee, Young Suk Shim

**Affiliations:** Department of Pediatrics, Ajou University Hospital, Ajou University School of Medicine, Suwon, Republic of Korea

**Keywords:** metabolic syndrome, parental influence, sex characteristics, metabolic diseases, risk factors, comparative studies

## Abstract

**Background:**

Metabolic syndrome (MetS) is known to have parental influence on children’s metabolic health, increasing the risk for the cardiometabolic diseases. However, the extent of the association and its sex-specific differences remain unclear.

**Objective:**

This retrospective, comparative study aimed to investigate the influence of parental MetS on their offspring, utilizing data from the Korea National Health and Nutritional Examination Survey.

**Methods:**

The study population was narrowed to 5,245 participants. Each sex was further divided into three groups: children with neither parent having MetS were labeled as “No,” those with only one affected parent was labeled as “Either,” and those with both parents affected were labeled as “Both.” Analysis of covariance and multiple regression analysis were used to compare the cardiometabolic risk factors among the three groups.

**Results:**

Children with one or both parents affected by MetS had significantly higher waist circumference, blood pressure, triglycerides, and fasting glucose levels. These associations were more profound in boys than in girls.

**Conclusion:**

Metabolic risk factors were more strongly associated with parental MetS in boys than in girls. The number of parental MetS cases appeared to have a proportional impact on metabolic components in boys, unlike the variable results observed in girls. These results emphasize the need for targeted interventions in families with a history of MetS.

## 1 Introduction

Over the past decades, the incidence of obesity has been increasing worldwide, regardless of sex, race, or age, and the related health risks have concomitantly increased in children and adolescents (1, 2). Metabolic syndrome (MetS), previously known as “Syndrome X,” is a major condition arising from children being obese, not only in developed countries but also in the urban population of developing countries (3, 4). In South Korea, a country that has undergone rapid westernization, the prevalence of MetS is reportedly 4.6% (5).

Importantly, MetS in childhood and adolescence is strongly associated with an increased risk of cardiovascular disease, type 2 diabetes mellitus, and premature mortality in adulthood (6, 7). Longitudinal cohort studies have demonstrated that children with persistently elevated metabolic risk factors are more likely to develop cardiometabolic complications later in life, even if they do not meet the full diagnostic criteria for MetS during childhood (8). Despite this well-established evidence of long-term risks, defining pediatric MetS remains challenging. The lack of universally accepted definition of MetS in children and adolescents is due to the diverse clinical characteristics of the disease and the difficulties of conducting extensive examinations in children (9, 10). A comprehensive understanding of MetS and its risk factors is mandatory to prevent its long-term cardiometabolic comorbidities, leading to early diagnosis and intervention (10).

Among the known risk factors of MetS, there are growing concerns regarding socioeconomic and familial factors. Various studies have suggested potential risk factors for adult MetS, such as weight gain, central adiposity, smoking, and sodium-rich and high-carbohydrate diet (11–13). In contrast, engaging in moderate to vigorous physical activity and consuming two to three cups of coffee daily may offer protective effects against MetS (14, 15). Since children tend to select lifestyle habits based on their parents, these findings emphasize the need for a familial approach to screening and early intervention for MetS in the pediatric population. Recently, a genetic correlation between MetS and cardiometabolic traits has been proposed, suggesting a potential basis for the influence of affected parents on their children (16). Additional evaluation of such genetic influences is necessary; however, there is currently insufficient data on the biological factors shared between parents and children. While familial and genetic influences on MetS have been suggested, it remains unclear whether the number of affected parents confers a cumulative risk on their offspring. Understanding this relationship is crucial for developing individualized prevention strategies.

Multiple studies have suggested sex-related differences in cardiometabolic risk factors in children, potentially due to differences in hormonal regulation, fat distribution, and oxidative stress responses (17, 18). Prepubertal girls have been shown to have higher levels of triglycerides (TG), total cholesterol (TC), and insulin resistance compared to boys, suggesting an increased susceptibility to metabolic syndrome (18). Additionally, pubertal development has been associated with fat accumulation and worse glucose metabolism, especially in girls (19). These sex-specific differences may influence how parental MetS affect metabolic outcomes in their offspring, but studies investigating such differential effects are limited.

This study aimed to ascertain the influence of parental MetS on children’s metabolic health, focusing on the additive impact of the number of affected parents and whether the influence differ by sex of the children. Given the increasing prevalence of childhood MetS and its cardiometabolic sequelae, understanding these familial and sex-specific influences is critical for developing precision preventive strategies.

## 2 Materials and methods

### 2.1 Study subjects

This retrospective study used data from the Korean National Health and Nutrition Examination Survey (KNHANES) conducted between 2007 and 2020. KNHANES is a nationally representative cross-sectional survey conducted by the Korea Disease Control and Prevention Agency, with all participants providing written informed consent when participating. The survey employs a complex, stratified, multistage probability sampling method to ensure that the database represents the general South Korean population.

Since this study exclusively used fully anonymized KNHANES data without identifiable information, additional informed consent was waived. The study received approval from the Institutional Review Board of Ajou University Hospital (AJOUIRB-EX-2024-446). Among the 113,091 subjects enrolled in the KNHANES database, we selected children and adolescents aged 10–18 years whose biological parents were present (*n*=7,395). Subjects without anthropometrical or laboratory data, whose parents do not have such data, and with serum TG levels ≥400 mg/dL were excluded, resulting in a final target population of 5,245 children (**Supplementary Figure 1**).

The study population was divided into two primary groups based on sex: boys (*n*=2,785) and girls (*n*=2,460). Each sex group was further subdivided into three categories according to the number of parents diagnosed with MetS: children with neither parent affected were classified as “No,” those with one affected parent as “Either,” and those with both parents affected as “Both.”

### 2.2 Data selection

We selected clinical data, such as systolic and diastolic blood pressure (BP), serum glucose, TC, TG, high-density lipoprotein cholesterol (HDL-C), and low-density lipoprotein cholesterol (LDL-C) levels, of the subjects and their parents from KNHANES database. Other clinical data included a diagnosis of type 2 diabetes mellitus (T2DM), hypertension, and dyslipidemia. Anthropometric data, including height, weight, waist circumference (WC), and body mass index (BMI) were selected and converted into standard deviation scores (SDS) to facilitate their use in statistical applications. Demographic data known to affect MetS, including alcohol consumption, smoking, physical activity, rural residence, and household income, were also collected. Although dietary intake data are available in KNHANES, we excluded them from our models due to their limited reliability in children, a high rate of missing values, and the potential for measurement bias from proxy-reported responses.

### 2.3 Definition of MetS and cardiometabolic factors

Metabolic syndrome in children and adolescents was defined using a modified version of the National Cholesterol Education Program Adult Treatment Panel III criteria, adapted for pediatric populations as proposed by Cook et al. (20). MetS was defined as three or more of the following five cardiometabolic criteria: elevated WC, BP, glucose, and TG, or reduced HDL-C. Each category was defined based on the deviations from normal values according to age and sex in the Korean population. Elevated WC was identified when WC was at or above the 90th percentile, specific to sex and age. BP was considered elevated if the systolic BP (SBP) or diastolic BP (DBP) was at or above the 90th percentile, adjusted for sex, age, and height, based on the 2017 Korean National Growth Charts for children and adolescents (17), or if the subjects were undergoing treatment for hypertension. Elevated glucose levels were determined by fasting glucose measurements ≥110 mg/dL or by a prior diagnosis of T2DM. TG was considered elevated when fasting TG levels were ≥110 mg/dL and HDL-C was considered reduced when its levels were < 40 mg/dL.

### 2.4 Statistics

We used the R version 4.1.1 for Windows (R Foundation for Statistical Computing, Vienna, Austria) for statistical analyses. Independent t-tests, analysis of variance, and chi-square tests were performed to compare the clinical characteristics of the study population and their parents by sex. Comparative analyses were performed for each sex by subgroups based on the number of affected parents. Continuous and categorical variables were presented as mean ± standard deviation and numbers ± percentages, respectively. Additionally, analysis of covariance (ANCOVA) was performed to estimate the adjusted means of the cardiometabolic risk factors between the subgroups after adjusting for age, alcohol consumption, smoking, rural residence, household income, parental age, and parental BMI. The adjusted odds ratio (aOR) and 95% confidence intervals of MetS incidence and its components were calculated using multiple logistic regression analysis after adjusting for age, sex, alcohol consumption, smoking, rural residence, household income, parental age, and parental BMI.

## 3 Results

### 3.1 Clinical characteristics of the target population

The clinical characteristics of the participants (n=5,245) are shown in **Table 1**. Among them, 2,785 boys and 2,460 girls showed significant differences in mean values of SDS of height and weight, SBP, serum glucose, TC, HDL-C, and LDL-C levels, percentage of smokers, and physical activities with a *p*-value of <0.001. DBP and the rate of alcohol consumption were also significantly different between boys and girls (*p*-value=0.003). In most aspects, boys tended to have higher mean values, except for TC, HDL-C, and LDL-C levels.

**Table 1:** Clinical characteristics of the study population by gender groups.

	Boys (n=2,785)	Girls (n=2,460)	P-value
Age (years)	13.72 ± 2.53	13.83 ± 2.53	0.113
Height SDS	0.58 ± 1.07	0.43 ± 1.04	<0.001
Weight SDS	0.33 ± 1.21	0.22 ± 1.11	<0.001
WC SDS	–0.20 ± 1.13	–0.18 ± 1.11	0.529
BMI SDS	0.07 ± 1.29	0.02 ± 1.19	0.095
Systolic BP (mmHg)	108.80 ± 10.68	104.20 ± 9.06	<0.001
Diastolic BP (mmHg)	66.25 ± 9.87	65.52 ± 8.07	0.003
Glucose (mg/dL)	90.94 ± 6.81	89.49 ± 9.91	<0.001
TC (mg/dL)	156.47 ± 26.95	163.40 ± 26.10	<0.001
TG (mg/dL)	82.43 ± 46.76	84.69 ± 41.92	0.065
HDL–C (mg/dL)	50.10 ± 9.90	52.58 ± 10.01	<0.001
LDL–C (mg/dL)	89.88 ± 23.25	93.88 ± 22.73	<0.001
Alcohol drinker	689 (24.7%)	521 (21.2%)	0.003
Smoker	353 (12.7%)	124 (5.0%)	<0.001
Physical activity	1026 (36.8%)	774 (31.5%)	<0.001
Rural residence	455 (16.3%)	405 (16.5%)	0.932
Household income ≤ first quartile	130 (4.7%)	121 (4.9%)	0.719
T2DM diagnosis	1 (0.1%)	0 (0%)	NC
Hypertension diagnosis	0 (0%)	0 (0%)	NC
Dyslipidemia diagnosis	0 (0%)	0 (0%)	NC

SDS, standard deviation score; WC, waist circumference; BMI, body mass index; BP, blood pressure; TC, total cholesterol; TG, triglycerides; HDL-C, high-density lipoprotein cholesterol; LDL-C, low-density lipoprotein cholesterol; T2DM, type-2 diabetes mellitus; NC, not comparable.

The fathers of the boys were younger (*p*=0.002), had a higher WC (*p*=0.029), and were more physically active (*p*=0.024) than the fathers of the girls (**Supplementary Table 1**). Meanwhile, the mothers of the boys were older (*p*=0.002), had lower mean BMI and serum TC levels (*p=*0.032 and 0.041), higher mean SBP and DBP (*p*=0.029 and 0.011), and were diagnosed with T2DM more often (*p*=0.009) (**Supplementary Table 2**). Other clinical characteristics of the parents showed no significant differences between the boys and girls (**Supplementary Tables 1**, **2**).

### 3.2 Comparison analysis between the subgroups

**Figure 1:**
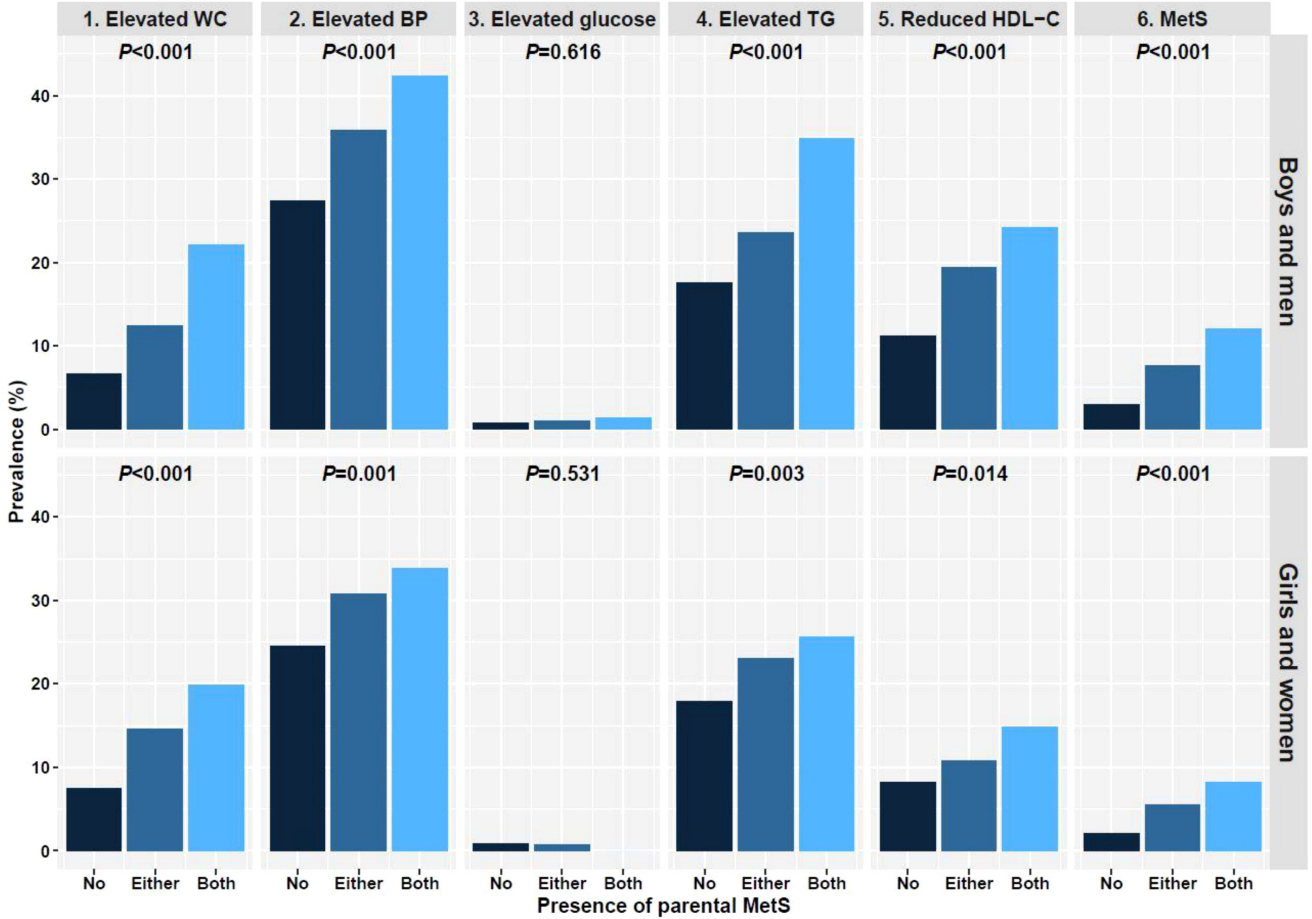
Cardiometabolic risk factors and the diagnosis of MetS in each subgroup.

**Table 2** shows a comparative analysis of the clinical parameters between the subgroups in each sex. In boys, significant differences were noted in SDS of weight, WC, and BMI, SBP, DBP, serum glucose, TC, TG, HDL-C, and LDL-C levels, and the percentages of elevated WC, BP, and TG, reduced HDL-C, and MetS diagnoses within the three subgroups with a *p*-value <0.001. In girls, SDS of weight, WC, and BMI, SBP, DBP, serum TG and HDL-C levels, percentage of elevated WC and BP, and MetS diagnosis were significantly different among the subgroups (*p*<0.001). Differences were also noted in Height SDS (*p*=0.039), LDL-C levels (*p*=0.021), percentage of alcohol drinkers (*p*=0.029), elevated TG levels (*p*=0.003), and reduced HDL-C levels (*p*=0.014) among girls. However, in girls, there were no significant differences in the mean serum glucose and TC levels (*p*=0.719 and 0.359, respectively) among the subgroups. Most parameters, except for elevated glucose, tended to increase significantly with the number of affected parents in both sexes (**Figure 1**). Only elevated glucose levels resembled sex differences, with girls tending to have lower serum glucose levels as the number of affected parents increased; however, the differences were not statistically significant (*p*=0.531).

**Table 2:** Comparison of the clinical parameters and the diagnosis of MetS among subgroups.

	Boys				Girls			
	No (n=1,641)	Either (n=995)	Both (n=149)	P-value	No (n=1,458)	Either (n=871)	Both (n=121)	P-value
Age (years)	13.54 ± 2.53	13.96 ± 2.50	14.21 ± 2.56	<0.001	13.71 ± 2.53	13.92 ± 2.51	14.70 ± 2.53	<0.001
Height SDS	0.54 ± 1.06	0.63 ± 1.07	0.68 ± 1.19	0.068	0.39 ± 1.03	0.47 ± 1.06	0.59 ± 1.08	0.039
Weight SDS	0.17 ± 1.16	0.52 ± 1.22	0.82 ± 1.40	<0.001	0.07 ± 1.05	0.39 ± 1.16	0.80 ± 1.16	<0.001
WC SDS	–0.37 ± 1.09	–0.01 ± 1.13	0.36 ± 1.23	<0.001	–0.34 ± 1.05	–0.00 ± 1.13	0.44 ± 1.20	<0.001
BMI SDS	–0.10 ± 1.23	0.28 ± 1.30	0.63 ± 1.46	<0.001	–0.15 ± 1.11	0.20 ± 1.24	0.64 ± 1.28	<0.001
Systolic BP (mmHg)	107.65 ± 10.52	110.03 ± 10.53	113.15 ± 11.36	<0.001	103.57 ± 8.86	104.82 ± 9.22	107.41 ± 9.33	<0.001
Diastolic BP (mmHg)	65.51 ± 9.79	67.11 ± 9.73	68.76 ± 10.76	<0.001	64.98 ± 7.90	66.25 ± 8.20	66.83 ± 8.62	<0.001
Glucose (mg/dL)	90.53 ± 6.81	91.43 ± 6.79	92.25 ± 6.62	<0.001	89.36 ± 11.42	89.66 ± 7.16	89.83 ± 6.78	0.719
TC (mg/dL)	155.18 ± 26.13	157.44 ± 27.23	164.11 ± 32.10	<0.001	163.00 ± 25.94	163.65 ± 26.49	166.42 ± 25.16	0.359
TG (mg/dL)	77.69 ± 43.39	86.86 ± 48.93	105.19 ± 57.31	<0.001	82.22 ± 39.52	87.75 ± 44.17	92.69 ± 50.72	0.001
HDL–C (mg/dL)	51.27 ± 9.88	48.72 ± 9.77	46.40 ± 8.87	<0.001	53.40 ± 10.01	51.70 ± 10.13	49.02 ± 7.69	<0.001
LDL–C (mg/dL)	88.38 ± 22.57	91.35 ± 23.35	96.67 ± 27.86	<0.001	93.15 ± 22.54	94.40 ± 22.87	98.86 ± 23.58	0.021
Alcohol drinker	381 (23.22%)	259 (26.03%)	49 (32.89%)	0.016	298 (20.30%)	186 (21.35%)	37 (30.58%)	0.029
Smoker	192 (11.70%)	138 (13.87%)	23 (15.44%)	0.156	76 (5.18%)	42 (4.82%)	6 (4.96%)	0.93
Physical activity	598 (36.44%)	363 (36.48%)	65 (43.62%)	0.211	444 (30.25%)	288 (33.07%)	42 (34.71%)	0.267
Rural residence	266 (16.21%)	160 (16.08%)	29 (19.46%)	0.568	241 (16.42%)	141 (16.19%)	23 (19.01%)	0.733
Household income ≤ first quartile	65 (3.96%)	54 (5.43%)	11 (7.38%)	0.061	71 (4.84%)	44 (5.05%)	6 (4.96%)	0.973
Elevated WC	108 (6.58%)	123 (12.36%)	33 (22.15%)	<0.001	109 (7.43%)	127 (14.58%)	24 (19.83%)	<0.001
Elevated BP	450 (27.42%)	357 (35.88%)	63 (42.28%)	<0.001	361 (24.59%)	268 (30.77%)	41 (33.88%)	0.001
Elevated glucose	12 (0.73%)	10 (1.01%)	2 (1.34%)	0.616	13 (0.89%)	6 (0.69%)	0 (0%)	0.531
Elevated TG	289 (17.61%)	235 (23.62%)	52 (34.90%)	<0.001	262 (17.85%)	201 (23.08%)	31 (25.62%)	0.003
Reduced HDL–C	184 (11.21%)	193 (19.40%)	36 (24.16%)	<0.001	121 (8.24%)	94 (10.79%)	18 (14.88%)	0.014
MetS	49 (2.99%)	76 (7.64%)	18 (12.08%)	<0.001	30 (2.04%)	48 (5.51%)	10 (8.26%)	<0.001

SDS, standard deviation score; WC, waist circumference; BMI, body mass index; TC, total cholesterol; TG, triglycerides; HDL-C, high-density lipoprotein cholesterol; LDL-C, low-density lipoprotein cholesterol; MetS, metabolic syndrome; BP, blood pressure.

Additional comparisons of cardiometabolic risk factors between each subgroup were performed, adjusting for the age of the subjects and their parents, parental BMI, percentage of alcohol consumption, smoking, rural residence, and household income. The results of these comparisons are presented in **Table 3**, with statistical significance in between subgroups and between “No” and “Either,” between “No” and “Both,” and between “Either” and “Both” subgroups – superscripted letters ‘a’ to ‘c,’ respectively. In both sexes, WC SDS significantly differed between the subgroups “No” and “Either,” whereas BMI SDS showed no statistically significant difference between subgroups. In boys, SBP showed substantial differences between the “Both” subgroup and the “No” and “Either” subgroups, but not between the “No” and “Either” subgroups. All subgroups showed significant differences among the girls. For DBP, boys showed significant differences only between the “No” and “Both” subgroups, while girls showed significant differences only between the “No” and “Either” subgroups. Serum glucose levels significantly differed between the “No” and “Either” subgroups in boys only. As for lipid profiles, most factors showed significant differences between subgroups in boys, except for the TC level in between “No” and “Either” subgroups and the HDL-C level in between “Either” and “Both” subgroups. In girls however, significant differences were found in serum TG and HDL-C levels between “No” and “Either,” and in serum TG, HDL-C, and LDL-C levels between “No” and “Both” subgroups.

**Table 3:** Comparisons between adjusted means of cardiometabolic risk factors in between subgroups.

	Boys			Girls		
	No (n=1,641)	Either (n=995)	Both (n=149)	No (n=1,458)	Either (n=871)	Both (n=121)
WC SDS	–0.25 ± 0.03	–0.16 ± 0.04^a^	–0.03 ± 0.09	–0.21 ± 0.03	–0.18 ± 0.04^a^	0.03 ± 0.10
BMI SDS	0.06 ± 0.03	0.09 ± 0.04	0.11 ± 0.10	0.08 ± 0.03	0.10 ± 0.10	0.12 ± 0.10
Systolic BP (mmHg)	108.33 ± 0.25	109.20 ± 0.32	111.25 ± 0.81^b,c^	103.61 ± 0.24	104.81 ± 0.31^b^	107.12 ± 0.82^b,c^
Diastolic BP (mmHg)	65.87 ± 0.23	66.65 ± 0.30	67.88 ± 0.77^b^	64.97 ± 0.22	66.32 ± 0.28^b^	66.52 ± 0.74
Glucose (mg/dL)	90.58 ± 0.17	91.39 ± 0.22^a^	91.93 ± 0.57	89.47 ± 0.27	89.46 ± 0.35	89.82 ± 0.92
TC (mg/dL)	155.03 ± 0.68	157.59 ± 0.87	164.80 ± 2.22^b,c^	162.67 ± 0.72	164.03 ± 0.94	167.66 ± 2.48
TG (mg/dL)	77.44 ± 1.15	87.21 ± 1.47^a^	105.53 ± 3.78^b,c^	81.56 ± 1.12	88.65 ± 1.46^a^	94.20 ± 3.86^b^
HDL–C (mg/dL)	51.15 ± 0.24	48.82 ± 0.31^a^	46.94 ± 0.80^b^	53.26 ± 0.26	51.79 ± 0.34^a^	50.05 ± 0.91^b^
LDL–C (mg/dL)	88.39 ± 0.58	91.32 ± 0.75^a^	96.75 ± 1.92^b,c^	93.10 ± 0.62	94. 50 ± 0.81	98.77 ± 2.15^b^

a Significant difference was determined using ANCOVA when the p-value was < 0.05 between “No” and “Either” subgroups.

b Significant difference was determined using ANCOVA when the p-value was < 0.05 between “No” and “Both” subgroups.

c Significant difference was determined using ANCOVA when the p-value was < 0.05 between “Either” and “Both” subgroups.

SDS, standard deviation score; BMI, body mass index; TC, total cholesterol; HDL-C, high-density lipoprotein cholesterol; LDL-C, low-density lipoprotein cholesterol; BP, blood pressure.

### 3.3 Adjusted odds ratios of the cardiometabolic components and the MetS

The aORs of MetS and its components based on the number of parents with MetS are organized in **Table 4**, with the statistical significance of the aOR compared to the reference – “No” subgroup – presented with an asterisk (*). Having one parent with MetS significantly increased the aOR of elevated BP, elevated TG, reduced HDL-C, and MetS compared to those without MetS (aORs = 1.39, 1.46, 1.61, and 2.11, respectively) in the study population. When both parents had MetS, the aORs for these components were significantly greater than in those with no or one parent affected (aORs = 1.68, 2.18, 1.99, and 2.63, respectively). The aORs for increased WC were higher in both subgroups, but were not statistically significant; the aOR for elevated glucose showed a decreasing trend as the number of affected parents increased.

**Table 4:** Adjusted odds ratios and confidence intervals of MetS in children and its components.

	No	Either	Both
All participants			
Elevated WC	Reference	1.37 (0.98–1.92)	1.79 (0.98–3.26)
Elevated BP	Reference	1.39 (1.20–1.60)*	1.68 (1.27–2.24)*
Elevated glucose	Reference	0.81 (0.40–1.66)	0.75 (0.16–3.53)
Elevated TG	Reference	1.46 (1.24–1.72)*	2.18 (1.59–2.98)*
Reduced HDL–C	Reference	1.61 (1.32–1.96)*	1.88 (1.30–2.70)*
MetS	Reference	2.11 (1.48–3.00)*	2.63 (1.47–4.68)*
Boys and men			
Elevated WC	Reference	1.23 (0.90–1.66)	1.62 (0.99–2.65)
Elevated BP	Reference	1.32 (1.09–1.61)*	1.70 (1.15–2.49)*
Elevated glucose	Reference	1.07 (0.42–2.76)	1.39 (0.26–7.48)
Elevated TG	Reference	1.52 (1.21–1.90)*	2.82 (1.86–4.30)*
Reduced HDL–C	Reference	1.89 (1.47–2.43)*	2.36 (1.49–3.76)*
MetS	Reference	2.20 (1.40–3.45)*	3.20 (1.52–6.47)*
Girls and women			
Elevated WC	Reference	1.30 (0.96–1.76)	1.21 (0.70–2.08)
Elevated BP	Reference	1.45 (1.17–1.79)*	1.74 (1.13–2.67)*
Elevated glucose	Reference	0.59 (0.19–1.85)	0.00 (0.00–Infinite)
Elevated TG	Reference	1.39 (1.09–1.76)*	1.67 (1.03–2.71)*
Reduced HDL–C	Reference	1.19 (0.86–1.66)	1.31 (0.71–2.42)
MetS	Reference	1.82 (1.05–3.32)*	2.11 (0.82–5.43)

*Significance was determined when the aOR compared to the reference when the p-value was < 0.05.

WC, waist circumference; BP, blood pressure; TG, triglycerides; HDL-C, high-density lipoprotein cholesterol; MetS, metabolic syndrome.

Similar results were observed in boys. Compared to the “No” subgroup, the “Either” subgroup showed significant increases in the aORs for elevated BP, elevated TG, reduced HDL-C, and MetS (aORs = 1.32, 1.52, 1.89, and 2.20, respectively). In the “Both” subgroup, the aORs for these components were significantly increased (aORs = 1.70, 2.82, 2.36, and 3.20, respectively) as the number of affected parents increased. In the overall study population, no significant increases in the aORs for increased WC or glucose levels were observed; only increasing trends were noted.

The results differed in girls, as significant increases in the aORs for the “Either” subgroup were observed only for elevated BP, elevated TG, and MetS (aORs = 1.45, 1.39, and 1.83, respectively). In the “Both” subgroup, only the aORs for elevated BP and TG were significantly increased (aORs = 1.74, and 1.67, each), again differing from the boys and the overall study population.

## 4 Discussion

This study demonstrated a dose-dependent association between the number of parents affected by MetS and the prevalence of MetS and its components in children, with the effect being more pronounced in boys. This finding emphasizes the clinical relevance of parental metabolic status and child sex as key stratifiers in early pediatric risk evaluation and intervention planning.

MetS, a cluster of interrelated cardiometabolic risk factors, has been associated with future cardiovascular and metabolic complications even in children (13, 21–23). Although its pathophysiology remains incompletely understood in pediatric populations, growing evidence suggests that early detection and intervention are critical to mitigate long-term damage (10). Bechard et al. suggested detrimental outcomes for hospitalized children with MetS, such as newly diagnosed cancer, aggravation of critical illnesses, or death (24). Moreover, longitudinal data indicate that children with persistent metabolic risk factors – even in the absence of a confirmed diagnosis of MetS – are more likely to develop subclinical inflammation and early cardiovascular changes, supporting the urgency of early screening (25).

From a clinical perspective, a familial approach is essential for managing children at risk of or diagnosed with MetS, as their lifestyle is directly inherited from their parents. Children learn and adopt their parents’ demographic characteristics, such as dietary habits and physical activities (11, 12, 26, 27). However, recent studies have suggested possible genetic traits inherited from parents that might increase the risk of cardiometabolic morbidity in children, indicating that parental metabolic status may influence offspring phenotypes beyond lifestyle, through mechanisms such as intrauterine programming, paternal epigenetic inheritance, and microbiome transmission (28, 29). Consistent with these findings, a dose-response relationship between the number of MetS affected parents and children’s MetS prevalence were verified in our study. The influence was examined after adjusting for socioeconomic and lifestyle confounders, supporting the heredity of MetS that are disparate from the behavioral features of parents. Although we adjusted for several lifestyle-related confounders such as physical activity, smoking, alcohol use, and socioeconomic variables, the association between parental MetS and children’s metabolic outcomes remained significant. This suggests that the familial influence extends beyond shared behavioral patterns and supports the contribution of genetic or epigenetic mechanisms. While we excluded dietary variables due to their limited reliability and high rates of missing or proxy-reported values in the pediatric dataset, future studies using standardized and validated metrics will be essential to further clarify the relative contributions of inherited or environmental factors.

We also observed significant sex-specific differences in the impact of parental MetS. Although MetS prevalence increased in both sexes with more affected parents, the association was more pronounced in boys. This result is consistent with those of previous studies that described a higher prevalence of MetS in boys than in girls (30, 31). Choi et al. reported an additional age-dependent difference in MetS pervasiveness by children’s sex; MetS was more prevalent in girls before 12 years of age and in boys after 13 years of age (30). We performed an additional comparison of MetS prevalence in the three subgroups by age in each sex, and the trend was consistent with that of a previous study. Girls showed a lower prevalence after 13 years of age, whereas boys showed a relatively higher prevalence (**Supplementary Figures 2**, **3**). The influence of affected parents seemed to be stronger in younger girls, despite the lack of statistical support (**Supplementary Figure 2**). Considering the pubertal hormonal changes in girls, this effect may be due to the protective effect of estrogen. Previous studies have demonstrated that estrogens increases insulin sensitivity and decreases the risk of central obesity (32, 33). Another potential mechanism that explains estrogen’s protective effect is that it suppresses metabolic inflammation by G-protein coupled estrogen receptor signaling (34).These mechanisms may underlie the lower susceptibility observed in girls, particularly during puberty. However, direct evidence for estrogen’s protective role in the inheritance of MetS is limited, warranting future studies including hormonal measurements and pubertal staging. Conversely, boys tend to accumulate more visceral fat and exhibit unfavorable lipid and blood pressure profiles during puberty, likely increasing their susceptibility to inherited cardiometabolic risks (35, 36). In addition, sex-specific behaviors may contribute to differential expression of familial risk.

Regarding individual components, our study found that elevated BP and TG were most strongly associated with parental MetS. This is consistent with prior studies identifying high heritability for these traits, independent of child BMI (37). Low HDL-C was also more pronounced in boys with affected parents, which may reflect a combination of genetic predisposition and androgenic effects on lipid metabolism (38). In contrast, the aORs of elevated WC showed insignificant increases by subgroup in both sexes, suggesting that the influence of parental MetS on central adiposity is trivial. This result differs from those of previous studies that reported a family history of obesity and cardiometabolic diseases as risk factors for childhood obesity (39). One explanation may be that parental BMI, rather than MetS status by itself, exerts a stronger influence on offspring adiposity. Additionally, children may already demonstrate dysmetabolic features prior to the onset of overt obesity, aligning with the concept of metabolically unhealthy normal-weight phenotypes. Further comprehensive studies on dietary habits and genetic aspects of central adiposity are required.

This study utilized a large, nationally representative dataset with detailed parent-child dyadic information, enhancing its generalizability and analytic power. We first discovered a proportionally increasing risk of MetS prevalence according to the number of affected parents and were able to demonstrate sex-based differences in the familial influence of MetS and its cardiometabolic factors. These results highlight the need for a familial approach in the clinical field, especially for boys and their parents with cardiometabolic conditions. Overall, the metabolic aspects in boys were more strongly associated with parental MetS than those in girls. The number of parents with MetS seemed to affect boys’ metabolic components proportionally compared to the variable results shown in girls. Clinically, these insights suggest that boys with both parent affected by MetS may benefit from earlier and more frequent metabolic screening.

In conclusion, the number of parents with MetS significantly influences the prevalence and severity of metabolic syndrome in children, with boys being particularly vulnerable. Our findings emphasize the need for sex-informed, family-based preventive strategies in pediatric care. Future longitudinal studies incorporating pubertal staging, hormone levels, and genetic markers are warranted to delineate causal pathways and optimize the timing of intervention.

## Data Availability

The original contributions presented in this study are included in the article/**Supplementary Material**. Further inquiries can be directed to the corresponding author.
