# Supplementary material for: No, either or both parents with metabolic syndrome: comparative study of its impact on sons and daughters
Source: Front Endocrinol (Lausanne). 2025 Apr 17;16:1518212. doi: 10.3389/fendo.2025.1518212 (PMC12043446; doi:10.3389/fendo.2025.1518212)
Supplement: Supplementary file 1 [file DataSheet1.docx]

Supplementary Material

# Supplementary Figures and Tables

## Supplementary Figures

#
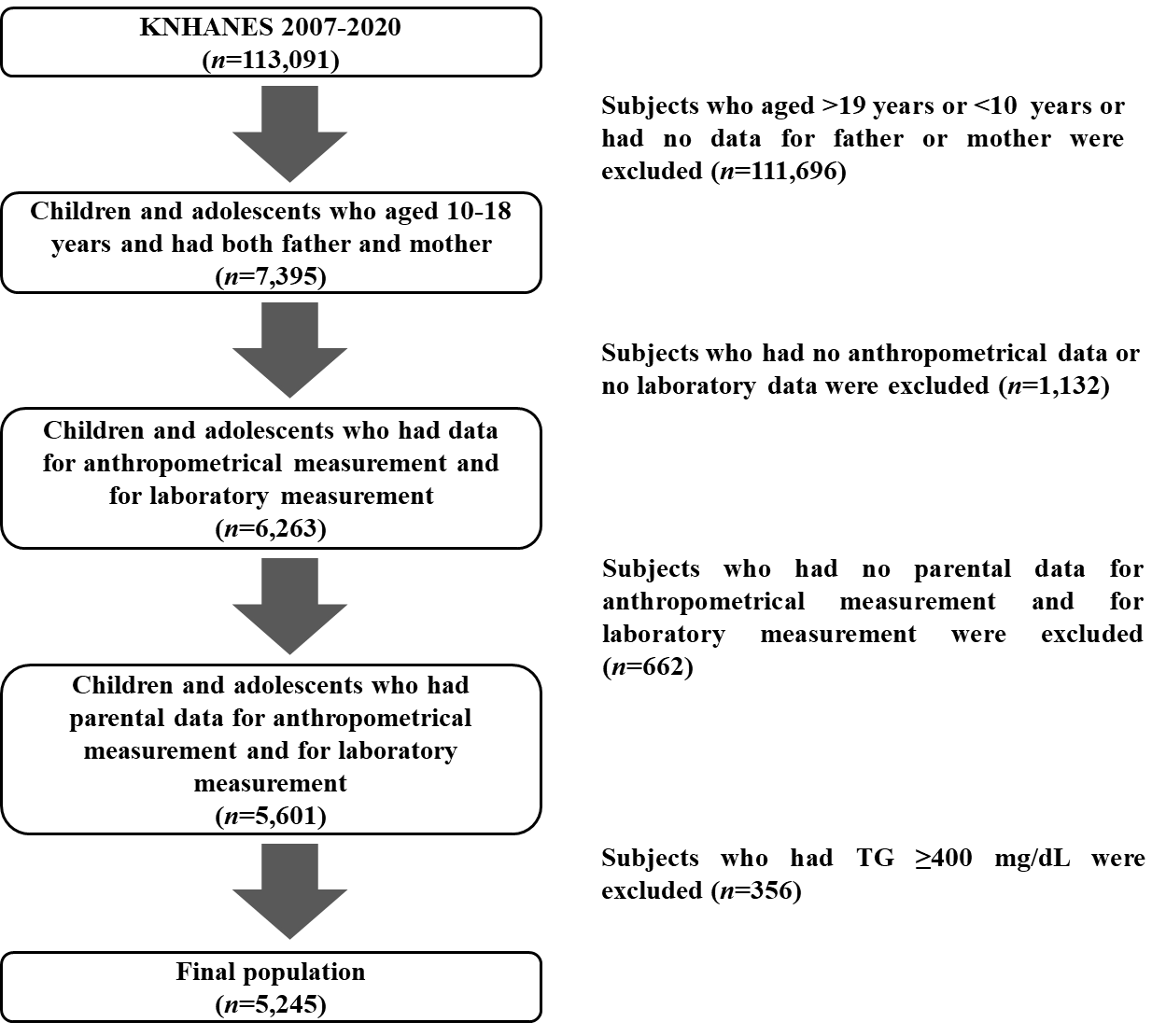


# Supplementary Figure 1. The flowchart of the study population.

**
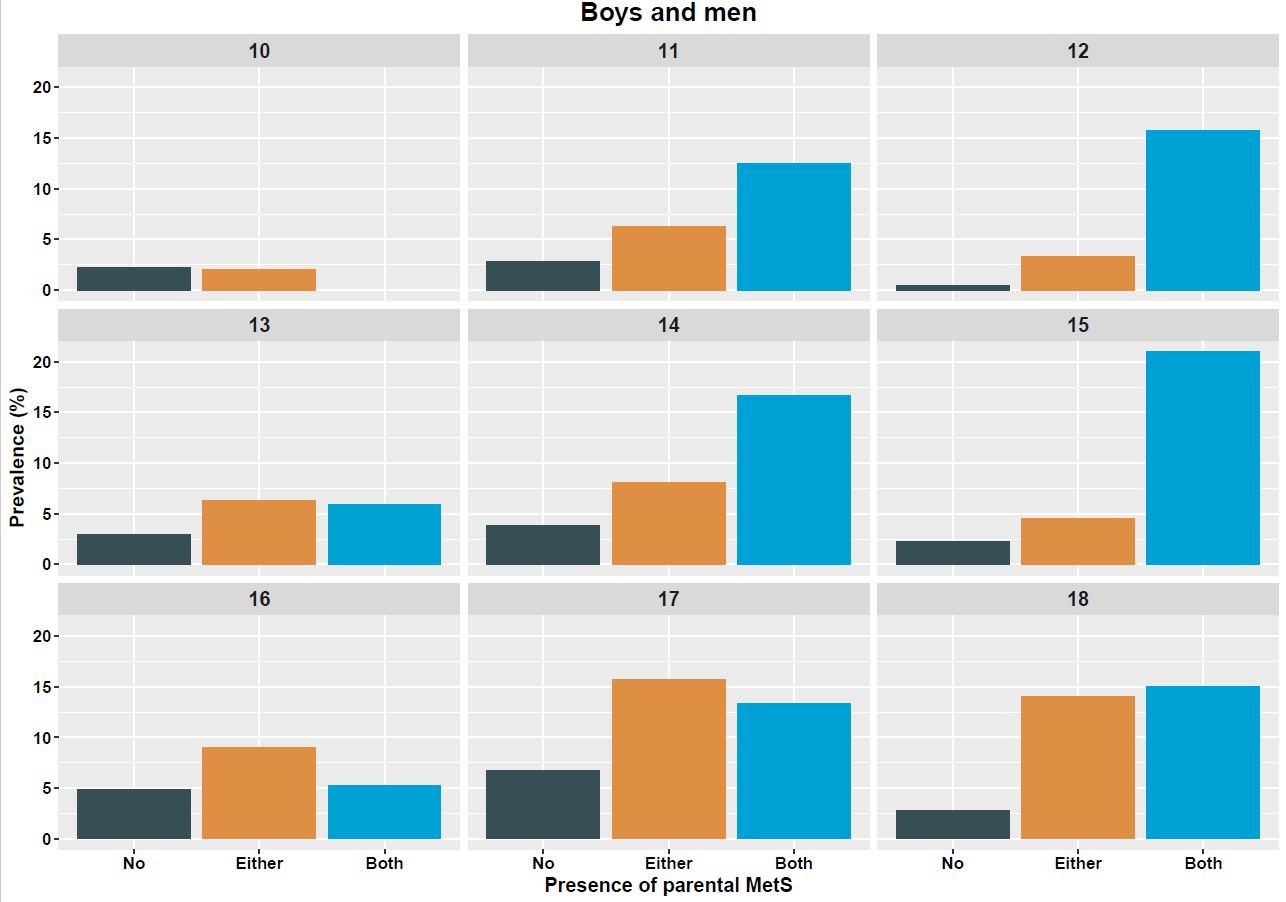
**

**Supplementary Figure 2.** Prevalence of MetS according to parental MetS in boys by their ages


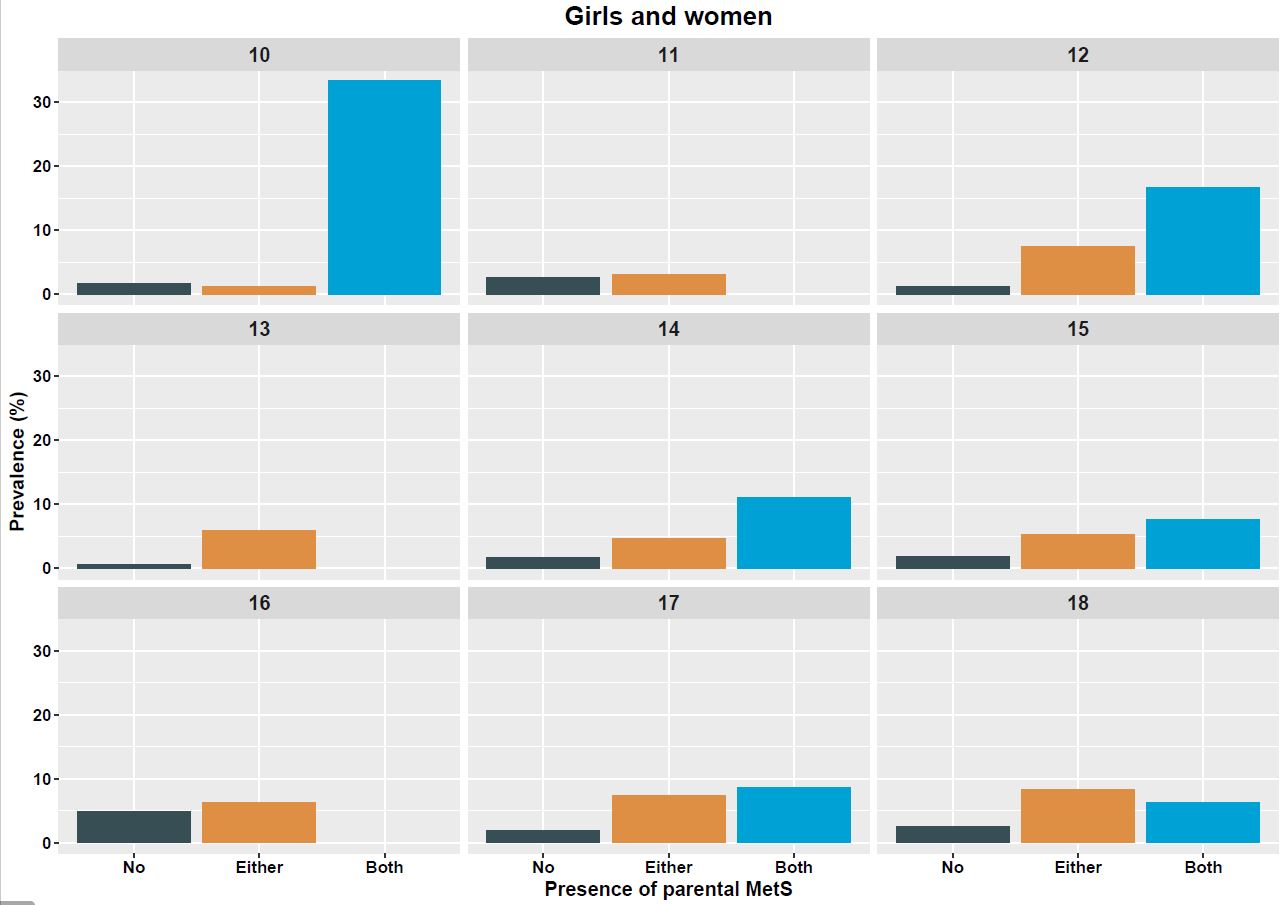


**Supplementary Figure 3.** Prevalence of MetS according to parental MetS in girls by their ages

## Supplementary Tables

**Supplementary Table 1.** Clinical characteristics of the fathers

|  | **Boys (n=2,785)** | **Girls (n=2,460)** | **P-value** |
| --- | --- | --- | --- |
| Age (years) | 45.80 ± 4.56 | 45.41 ± 4.54 | 0.002 |
| Height (cm) | 171.44 ± 5.80 | 171.19 ± 5.78 | 0.116 |
| Weight (kg) | 72.82 ± 10.32 | 72.29 ± 10.48 | 0.063 |
| WC (cm) | 86.20 ± 8.41 | 85.69 ± 8.46 | 0.029 |
| BMI (kg/m^2^) | 24.74 ± 3.03 | 24.63 ± 3.10 | 0.192 |
| Systolic BP (mmHg) | 118.55 ± 14.10 | 118.55 ± 13.82 | 0.997 |
| Diastolic BP (mmHg) | 81.28 ± 10.32 | 81.22 ± 10.32 | 0.814 |
| Glucose (mg/dL) | 100.61 ± 20.73 | 100.96 ± 22.64 | 0.57 |
| T‒C (mg/dL) | 195.52 ± 35.27 | 194.63 ± 35.52 | 0.364 |
| TG (mg/dL) | 154.59 ± 78.12 | 152.78 ± 77.68 | 0.399 |
| HDL‒C (mg/dL) | 46.81 ± 10.82 | 46.74 ± 10.47 | 0.804 |
| LDL‒C (mg/dL) | 117.79 ± 32.90 | 117.34 ± 32.44 | 0.616 |
| Alcohol drinker (%) | 2661 (95.5%) | 2345 (95.3%) | 0.750 |
| Smoker (%) | 2195 (78.8%) | 1927 (78.3%) | 0.696 |
| Physical activity (%) | 1751 (62.9%) | 1471 (59.8%) | 0.024 |
| Rural residence (%) | 455 (16.3%) | 405 (16.5%) | 0.932 |
| Household income ≤ first quartile | 130 (4.7%) | 121 (4.9%) | 0.719 |
| T2DM diagnosis (%) | 146 (5.2%) | 122 (5.0%) | 0.688 |
| Hypertension diagnosis (%) | 363 (13.0%) | 307 (12.5%) | 0.576 |
| Dyslipidemia diagnosis (%) | 266 (9.6%) | 233 (9.5%) | 0.959 |
| MetS (%) | 913 (32.8%) | 793 (32.2%) | 0.695 |
| WC, waist circumference; BMI, body mass index; T-C, total cholesterol; TG, triglycerides; HDL-C, high-density lipoprotein cholesterol; LDL-C, low-density lipoprotein cholesterol; T2DM, type-2 diabetes mellitus; MetS, metabolic syndrome | | | |

**Supplementary Table 2.** Clinical characteristics of the mothers

|  | **Boys (n=2,785)** | **Girls (n=2,460)** | **P-value** |
| --- | --- | --- | --- |
| Age (years) | 42.85 ± 4.30 | 42.47 ± 4.33 | 0.002 |
| Height (cm) | 158.94 ± 5.37 | 158.84 ± 5.30 | 0.498 |
| Weight (kg) | 58.61 ± 8.61 | 59.06 ± 9.37 | 0.071 |
| WC (cm) | 77.28 ± 8.50 | 77.65 ± 9.11 | 0.128 |
| BMI (kg/m^2^) | 23.20 ± 3.19 | 23.39 ± 3.43 | 0.032 |
| Systolic BP (mmHg) | 111.22 ± 14.23 | 110.37 ± 13.78 | 0.029 |
| Diastolic BP (mmHg) | 73.93 ± 9.55 | 73.25 ± 9.57 | 0.011 |
| Glucose (mg/dL) | 94.76 ± 18.96 | 94.67 ± 19.68 | 0.874 |
| T‒C (mg/dL) | 187.44 ± 32.43 | 187.43 ± 31.88 | 0.992 |
| TG (mg/dL) | 97.97 ± 55.02 | 101.18 ± 58.57 | 0.041 |
| HDL‒C (mg/dL) | 54.32 ± 12.04 | 54.09 ± 12.00 | 0.489 |
| LDL‒C (mg/dL) | 113.53 ± 28.69 | 113.11 ± 28.28 | 0.593 |
| Alcohol drinker (%) | 2474 (88.8%) | 2221 (90.3%) | 0.095 |
| Smoker (%) | 160 (5.7%) | 156 (6.3%) | 0.397 |
| Physical activity (%) | 1765 (63.4%) | 1573 (63.9%) | 0.691 |
| Rural residence (%) | 455 (16.3%) | 405 (16.5%) | 0.932 |
| Household income ≤ first quartile | 130 (4.7%) | 121 (4.9%) | 0.719 |
| T2DM diagnosis (%) | 57 (2.0%) | 62 (2.5%) | 0.291 |
| Hypertension diagnosis (%) | 176 (6.3%) | 114 (4.6%) | 0.009 |
| Dyslipidemia diagnosis (%) | 129 (4.6%) | 96 (3.9%) | 0.218 |
| MetS (%) | 380 (13.6%) | 320 (13.0%) | 0.525 |
| WC, waist circumference; BMI, body mass index; T-C, total cholesterol; TG, triglycerides; HDL-C, high-density lipoprotein cholesterol; LDL-C, low-density lipoprotein cholesterol; T2DM, type-2 diabetes mellitus; MetS, metabolic syndrome | | | |
